# Unusual root morphology in second mandibular molar with a radix entomolaris, and comparison between cone-beam computed tomography and digital periapical radiography: a case report

**DOI:** 10.1186/s13256-015-0681-x

**Published:** 2015-09-22

**Authors:** Elisardo López-Rosales, Pablo Castelo-Baz, Roland De Moor, Manuel Ruíz-Piñón, Benjamín Martín-Biedma, Purificación Varela-Patiño

**Affiliations:** 1 Department of Operative Dentistry and Endodontics, School of Dentistry, University of Santiago de Compostela, C/ Entrerríos s/n Santiago Compostela, A Coruña, 15782 Spain; 2 Department of Operative Dentistry and Endodontology, Dental School, Ghent University, Ghent University Hospital, Gent, Belgium

## Abstract

**Introduction:**

Radix entomolaris presents with an unusual morphology and is a rare occurrence. It is mainly observed in mandibular first molars. The incidence varies in different populations but it is far from common. This is especially true for mandibular second molars which possess the lowest prevalence. Some case reports have shown the presence of this finding in mandibular second molars; however, cases of patients of a white background have not been reported.

**Case presentation:**

The diagnosis and treatment of an infected radix entomolaris in a mandibular second molar in a 45-year-old white man is presented. The diagnosis was made with standard endodontic techniques. Conventional radiographic imaging was augmented with cone-beam computed tomography scans and three-dimensional images which were constructed with dedicated software. The endodontic treatment was done using accepted endodontic procedures. Clinical and radiographic evidence of healing was seen after a 14-month follow-up.

**Conclusions:**

The implications of complex and unpredictable root anatomy are discussed in this report. The clinician should consider the possibility of encountering a mandibular second molar with a radix entomolaris. Cone-beam computed tomography is a useful tool in the diagnosis and improvement of root canal therapy.

**Electronic supplementary material:**

The online version of this article (doi:10.1186/s13256-015-0681-x) contains supplementary material, which is available to authorized users.

## Introduction

The success of root canal treatment requires in-depth knowledge of the anatomy and of the internal and external morphology of the treated teeth. The clinician should anticipate and identify the normality as well as the anatomical alterations that may be present because therapeutic failure may result from failure to identify alterations, such as supplementary roots or canals [1].

Carabelli [2] described a supernumerary root that was located on the distolingual area of mandibular molars and called it “radix entomolaris” (RE), referring to it as “radix paramolaris” (RP) when located in the mesiobuccal (MB) area. The prevalence of this root is directly associated with ethnic groups and geographical areas. The RE is mainly observed in mandibular first molars with an incidence between 5.8 and 33.1% in Asian populations and populations with Mongoloid features (Inuit and American Indians) [3–5], in Indian populations between 2.19 and 13.3% [6, 7], in Arab populations between 2.3 and 6.0% [8, 9], in Euro-Asians between 1.0 and 4.2% [3, 10], in African populations between 0.7 and 3.1% [11, 12], and, finally, with whites of the European continent (Spain, Germany and UK) exhibiting a prevalence of 0%, 0.7% and 3.3% respectively [13–15], and with whites of the American continent (USA and Brazil) exhibiting a prevalence of 2.2% and 4.2%, respectively [16, 17].

The most common radicular morphology of the mandibular second molar in the white population is two roots and three canals (two mesial canals and one distal). The most frequent anatomical alteration is a single root with one single oval canal [18]. It is the tooth with the lowest prevalence of RE, with percentages that vary from 0 to 1.3% [19, 20]. To date, cases of white patients with RE in mandibular second molar have not been reported.

This case report of a white patient presents endodontic treatment of a mandibular second molar with RE centrally located between the distal and mesial root components (TypeAC location, according to Carlsen and Alexandersen [21]), type III curvature, according to De Moor *et al.* [22] (curvature in the coronal third and buccal curvature from the middle third or apical third of the root) and type III radiographic, according to Wang *et al.* [23] (full overlapping from the coronal third to apical third), as well as the presence of four independent canals in which the diagnostic accuracy of cone-beam computed tomography (CBCT) and digital periapical radiography (PR) are compared.

## Case presentation

**Fig. 1:**
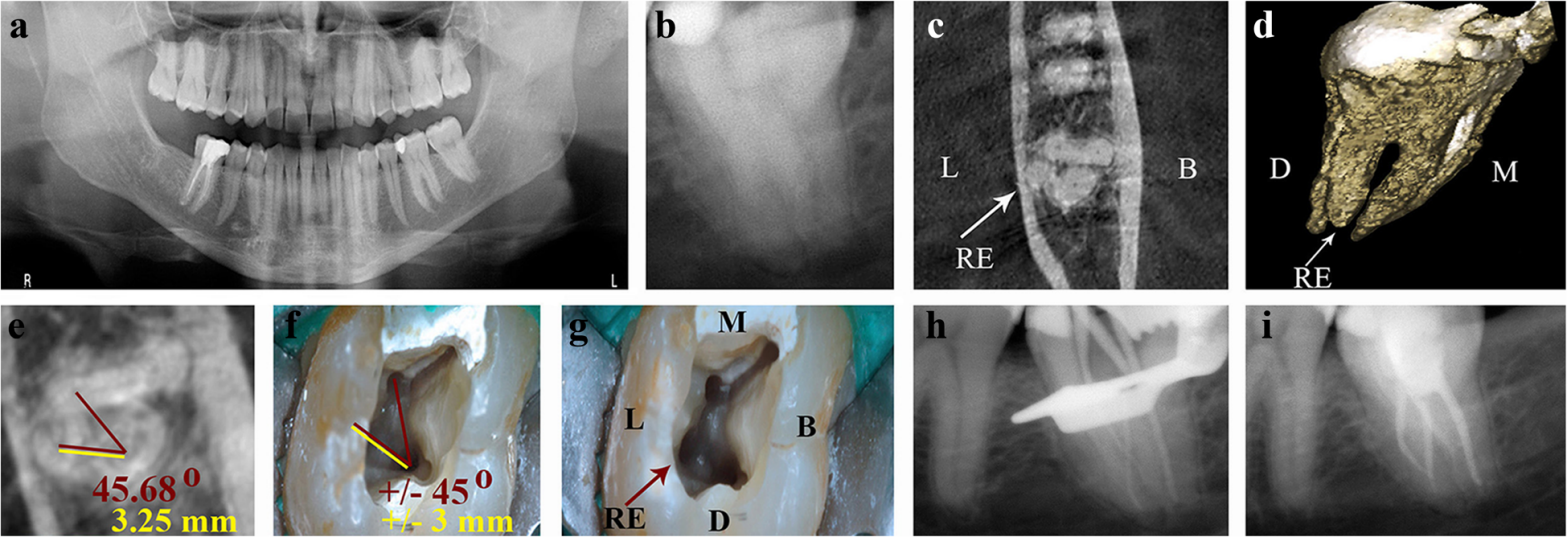
Preoperative and postoperative root canal therapy. **a** Orthopantomography. **b** Preoperative periapical radiograph of the tooth 3.7. A periapical radiolucent image around the distal root is observed. **c** Cone-beam computed tomography, axial view at the level of the middle third of the roots. *Arrow*: a third root is observed in the lingual area, radix entomolaris (*RE*) type, with a central location between the distal and mesial root components. **d** Cone-beam computed tomography, three-dimensional rendered image. The characteristics of the radix entomolaris (*RE*) are appreciated. *Arrow:* RE **e** Cone-beam computed tomography, axial view from the coronal third of the roots. *Red lines*: angle from the mesiolingual, distal and radix entomolaris canals. *Yellow line*: distance between the distal and radix entomolaris canals. **f** Pulp chamber floor of tooth 3.7 shown with the operating microscope (12×). The measures performed with the cone-beam computed tomography are transposed to a clinical setting. *Red lines*: angle from the mesiolingual, distal and radix entomolaris canals. *Yellow line*: distance between the distal and radix entomolaris canals. **g** *Arrow*: the orifice of the radix entomolaris (*RE*) is visualized. **h** Master cone radiograph. **i** Immediate postoperative radiograph. The four canals appear sealed Abbreviations, L: lingual; B: buccal; D: distal; M: mesial.

**Fig. 2:**
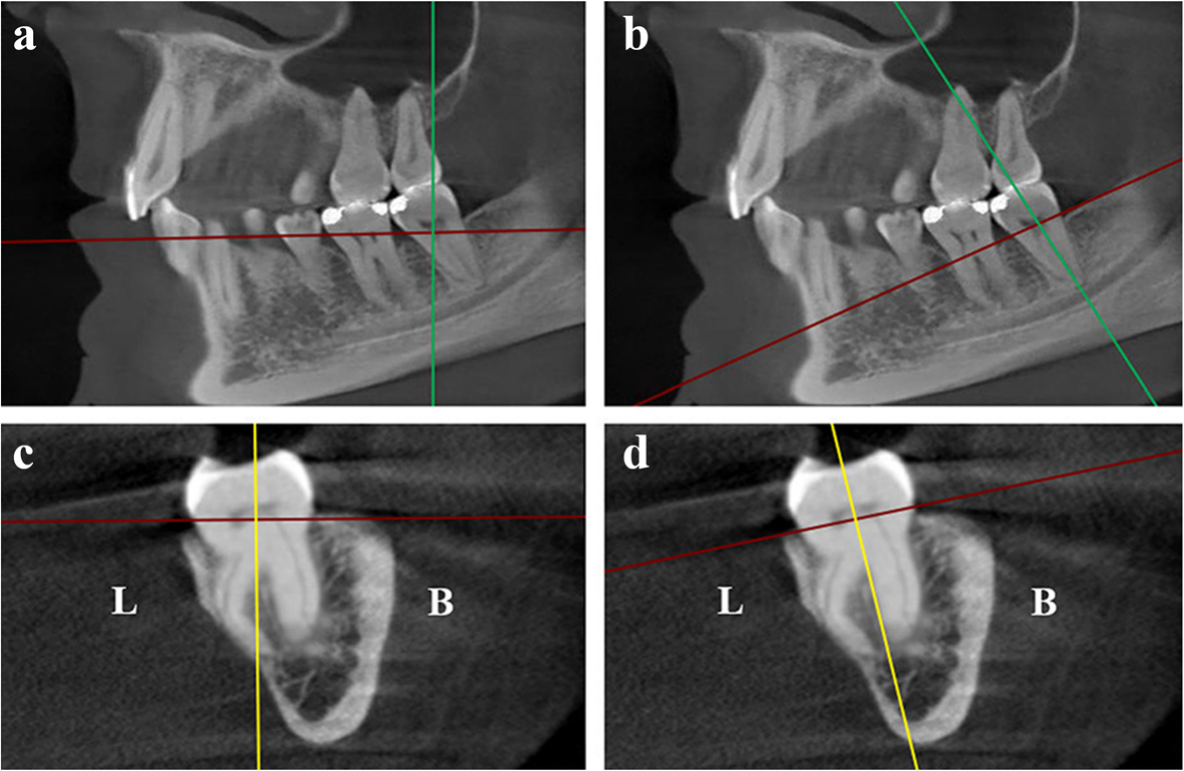
Cone-beam computed tomography. **a** Sagittal view. *Green line*: coronal axis. *Red line*: axial axis. The axes are incorrectly oriented with respect to tooth 3.7. **b** *Green line*: coronal axis. *Red line*: axial axis. The axes are correctly oriented (parallel and perpendicular to the longitudinal axis of the tooth 3.7). **c** Coronal view. *Yellow line*: sagittal axis. *Red line*: axial axis. The axes are oriented incorrectly regarding the tooth 3.7. **d** *Yellow line*: sagittal axis. *Red line*: axial axis. The axes are correctly oriented. Section shows radix entomolaris type III curvature Abbreviations, L:  lingual; B: buccal.

A 45-year-old white man was referred to our Endodontic department with intermittent acute pain of spontaneous onset in the left posterior-inferior area; the referring dentist commented that the symptoms started 2 months prior to performing a mesial-occlusal restoration (Class II) in the patient’s mandibular second molar. Tooth 3.7 was negative for the pulp vitality test (cold) and positive for the diagnostic tests (percussion and palpation). Signs of clinical inflammation were absent in the surrounding tissues. A 3.0 mm-diameter circumscribed radiolucent image around his distal root apex was observed on PR (RVG Satelec Sopix, Acteon Group, Marseilles-La Ciotat, France; Fig. 1b), which confirmed the diagnosis of symptomatic apical periodontitis due to pulp necrosis (according to the American Association of Endodontists, AAE, Consensus Conference Recommended Diagnostic Terminology of 2009). When examining the radicular morphology with the periapical and orthopantomography radiographs (Fig. 1a), overlapping images of the roots were observed which alerted us to the complex anatomy; the patient was referred for a CBCT (ProMax 3D Max, Planmeca, Helsinki, Finland). The sagittal and coronal axes from the dental imaging software (Romexis Viewer, Planmeca) were placed in parallel and the axial axis perpendicular to the longitudinal axis of the tooth 3.7 (Fig. 2). A horizontal cross-section (axial view) at the level of the middle third of the roots was performed, which revealed a third root located lingually and four independent canals (Fig. 1c). In the three-dimensional rendered image and coronal view, the characteristics of the RE could be detected. An independent root located on the lingual side of the tooth between the mesial and distal roots (RE type AC location), with a comparable slightly smaller length (Fig. 1d) and a buccally orientated curvature (type III curvature; Fig. 2) was seen. Having identified the root anatomy, the orifice of the RE in the pulp chamber floor was located; for this purpose, a horizontal cross-section (axial view) at the level of the coronal third of the roots was performed under the cementoenamel junction on the vertical cross-section viewed from the front (coronal view) and vertical cross-section viewed laterally (sagittal view; Fig. 2). The angle between the mesiolingual (ML) canal, the distal canal and RE was measured (45.68 degrees), as well as the interorifice distance between the RE and the distal canal (3.25mm; Fig. 1e).

**Fig. 3:**
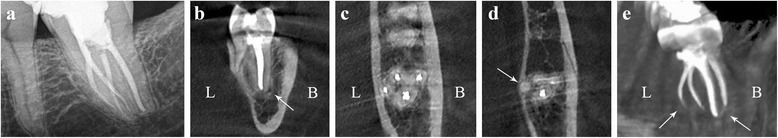
Fourteen-month postoperative follow-up. **a** Periapical radiography, complete bone remineralization of the periapical lesion of the distal root is observed. **b** Cone-beam computed tomography, coronal section. *Arrow*: partial bone remineralization of the previous lesion is observed. **c** Cone-beam computed tomography, axial view on the middle third of the roots. Obturation of the four canals is observed. **d** Axial view of the apical third of the roots (1mm from the apex). *Arrow*: the absence of sealing material in the radix entomolaris canal is observed. **e** Cone-beam computed tomography, three-dimensional rendered image. *Arrows*: radiopaque changes in the last millimeters of the mesiobuccal and radix entomolaris canals are observed Abbreviations, L: lingual; B: buccal.

An inferior alveolar nerve block followed by buccal infiltration was performed with 3.6mL of 2% lidocaine/epinephrine 1:80,000. The tooth was isolated under rubber dam (Hygenic Coltene, Ohio, USA) with a number 12a clamp and a number 6 Young metal frame (Hu-Friedy Mfg. Co., LLC, Chicago, USA). Access cavity preparation was created using a round diamond Endo-Access bur and an Endo-Z bur (Maillefer, Ballaigues, Switzerland). Upon visual inspection with an operating microscope (OPMI PROergo, Zeiss, Zaventem, Belgium) and an endodontic explorer (DG-16, Ash Instruments, Dentsply, Gloucester, UK), the orifices of the three canals, MB, ML and distal, were located. Using an ultrasonic tip with a non-cutting tip (Start-X number 1, Dentsply Maillefer) and considering the spatial location of the RE obtained with the CBCT (Fig 1e), the orifice to the canal was detected, by eliminating part of the lingual wall at +/− 45 degrees and +/− 3mm from the distal canal (Fig. 1f, g). The root canal length was determined using an electronic apex locator (Root ZX II, J. Morita MFG. Corp. Kyoto, Japan) and a pre-curved stainless steel K-File ISO number 10 (Dentsply Maillefer). The ISO 10 file also confirmed patency up to the apical foramen. The preflaring, the glide path and the root canal preparation were performed using nickel-titanium rotating files systems (PathFiles and ProTaper Universal, Dentsply Maillefer). Throughout the procedure, the canals were irrigated with abundant 5.25% sodium hypochlorite solution using a needle 0.30mm in diameter (NaviTip, Ultradent South Jordan, Utah, USA), and for a minimum of 30 minutes after the last file used. The final irrigation was performed with an ultrasonic irrigation system (ProUltra PiezoFlow, Dentsply, Tulsa, USA) for 1 minute for each canal with 10mL of sodium hypochlorite solution and thereafter for 1 minute with 17% ethylenediaminetetraacetic acid, subsequently leaving the solution passively for 5 minutes. The canals were dried with paper points of 4% conicity (R&S, Paris, France), and an endodontic cement was placed (Pulp Canal Sealer, SybronEndo, Orange, CA, USA). The obturation of the root canals was performed in the same session using Carrier-Based Thermoplasticized technique with a warm layer of alpha-phase gutta-percha (Thermafil, Dentsply Maillefer, Konstanz, Germany) (Fig. 1h, i). For adhesive sealing of the pulp chamber, the etch-and-rinse technique was performed with 37% phosphoric acid (Dentaflux, J. RIPOLL S.L.) and dentin adhesive (Adper Scotchbond, 3M ESPE, Minnesota, USA). The obturation was performed using flowable resin composite (Filtek Supreme, 3M ESPE) on the orifice of the canals and a light-curing provisional material was placed (Telio CS Onlay, Ivoclar Vivadent AG Bendererstrasse Schaan, Liechtenstein). The patient was referred to his general dentist for a permanent coronal restoration. After 14 months, postoperative control was performed and revealed negative results on the diagnostic clinical tests (percussion and palpation) and on the radiographic tests with a orthoradial PR (RVG Kodak 5100 Carestream Health, Rochester, NY, USA) performed with the Rinn set (Dentsply Rinn, Elgin, Illinois, USA) and the CBCT (Promax 3D Max, Planmeca; Fig. 3 Additional file 1).

## Discussion

Depending on the ethnic origin, RE in mandibular first molar may be a common anatomical alteration, as is the case with Asian patients [5], or an atypical morphology, as in white patients [14]. For the mandibular second molar, studies indicate that RE is a dysmorphic alteration (rare or unusual morphological alteration) even in ethnic populations with high rates of RE in mandibular first molars [20], and a third root in these teeth is linked more to the RP [24].

Alterations in the number of canals have been observed, such as those found by Pineda and Kuttler [25] in an “*in vivo*” study with patients of Mexican origin, revealing that 3% of mandibular second molars had four independent canals. Vertucci [26] reported, in an “*in vitro*” study, a 5% prevalence of these teeth with four canals in American populations of white ethnicity and, similarly, Walker [27] encountered a prevalence of 1% in the population of the south of China. Furthermore, cases with five (three in the mesial root and two in the distal root) and seven root canal systems (four in the mesial root and three in the distal root) have been reported [28, 29]. Although these anatomical aberrations are important alterations, most of these findings have been reported in mandibular second molars with only two roots. Alterations in number of roots have been observed in an “*in vivo*” study [20] with patients from China, revealing that 1.3% of mandibular second molars had three roots. Two cases with patients of Indian origin [30, 31] reported similar features (three roots, RE). Furthermore, a case (extracted mandibular second molar) with four roots in a patient from Sri Lanka [32] was reported. However, only one case with a patient of white origin with three roots (RP) has been reported [24]. Therefore, this is the first report of such a case (RE) in this population.

The majority of the orthoradial orientation of the PRs and the ability to distinguish individual REs became increasingly difficult because of the overlapping of the adjacent distobuccal root; for this, a 25-degree mesioradial orientation would be a better option in mandibular first molars [23]. Nonetheless, endodontic therapy in mandibular second molars is a more complicated issue than first molars due to the posterior position and consequent problems when taking proper PRs, eventually leading to gag reflex in the patient. For the case presented here, we faced these difficulties, and it was not possible to perform the initial PR with a mesioradial orientation.

The use of the CBCT on mandibular second molars is an excellent option to detect the anatomy and to mitigate the abovementioned inconveniences; the axial and coronal views and three-dimensional rendered images are a good option for a correct diagnosis and to observe the characteristics of the RE. CBCT, together with dental imaging software, allows us to measure the angles and distances with real values; thus, the spatial location of a structure, such as the entrance of the RE orifice in the pulp chamber, may be uncovered. It is important to align the long axis of the imaging plane with a specific anatomic structure for the values to be exact: taking the closest mesial root canal orifice to the radix as reference, helps to determine whether it is an entomolaris (ML) or a paramolaris (MB), thus forming an acute angle not greater than 90 degrees together with the distal root canal orifice and the radix for improved clinical interpretation (Fig. 1e). Today, precise instruments that transfer the information from the CBCT to a clinical setting are not available. However, the operating microscope offers increased visualization and light to use this information with approximately exact details (Fig. 1f, g).

Another aspect that should be highlighted is the short-, mid- and long-term postoperative controls that should be performed after endodontic treatment. The majority of the case reports indicate high-quality postoperative results but follow-ups performed using only PRs [22, 24]. A correct root canal treatment seen on the PR is not an absolute indicator of endodontic success. If we only consider the technique on its own, PRs offer inaccurate information on the situation compared with CBCT [33] because they provide a bi-dimensional image on the tri-dimensional structure of the teeth. As the canals are largely oval with the longer axis in the buccolingual or buccopalatal direction and the shorter axis in the mesiodistal direction, canal obturation can be difficult because PRs reveal only the shorter axis. In addition, PRs are more likely to miss apical periodontitis when it is still present [34]. This assumption is supported in this case, where postoperative PR control (14 months) revealed complete bone remineralization of the periapical lesion of the distal root (Fig. 3a), whereas a coronal section of the CBCT revealed partial remineralization of the lesion (Fig. 3b). Furthermore, the postoperative and control PRs revealed complete sealing of the four canals, whereas on an axial section of the CBCT (section 1mm from the radiographic apex), the RE is not sealed in the last millimeter of the canal (Fig. 3d). Finally, the three-dimensional rendered image (Fig. 3e) indicates a radiopaque change on the final millimeters of the MB and RE canals with low homogeneity of the gutta-percha in the area, which could be a consequence of the complex system of the radicular canals. Whilst this highlights the details observed with the CBCT in comparison to the periapical radiographic images, these findings did not affect the healing process.

## Conclusions

The implications of complex and unpredictable root anatomy are discussed in this report. The clinician should consider the possibility of encountering a mandibular second molar with three roots (RE). CBCT together with the operating microscope and ultrasonic tips are useful tools in the diagnosis and improvement of root canal therapy, providing greater security, predictability and overall efficiency when considering the technique, the biology and the clinical time needed.

## Consent

Written informed consent was obtained from the patient for publication of this Case report and any accompanying images. A copy of the written consent is available for review by the Editor-in-Chief of this journal.

## Additional file

Additional file 1: **Radix entomolaris in a mandibular second molar.** Three-dimensional rendered images. Postoperative follow-up. (MP4 1887 kb)

## Abbreviations

CBCT: Cone-beam computed tomography
MB: Mesiobuccal
ML: Mesiolingual
PR: Periapical radiography
RE: Radix entomolaris
RP: Radix paramolaris
